# Supplementary material for: Causal interplay between lactose intolerance and gut microbiota: a combined bidirectional Mendelian randomization and in vivo validation study
Source: Front Nutr. 2026 Jun 1;13:1803337. doi: 10.3389/fnut.2026.1803337 (PMC13265576; doi:10.3389/fnut.2026.1803337)
Supplement: Supplementary file 1 [file Data_Sheet_1.zip › supplementary materials/Forward/leave one out cross validation/ebi-a-GCST90027479.finngen_R12_E4_LACTONAS.pdf]

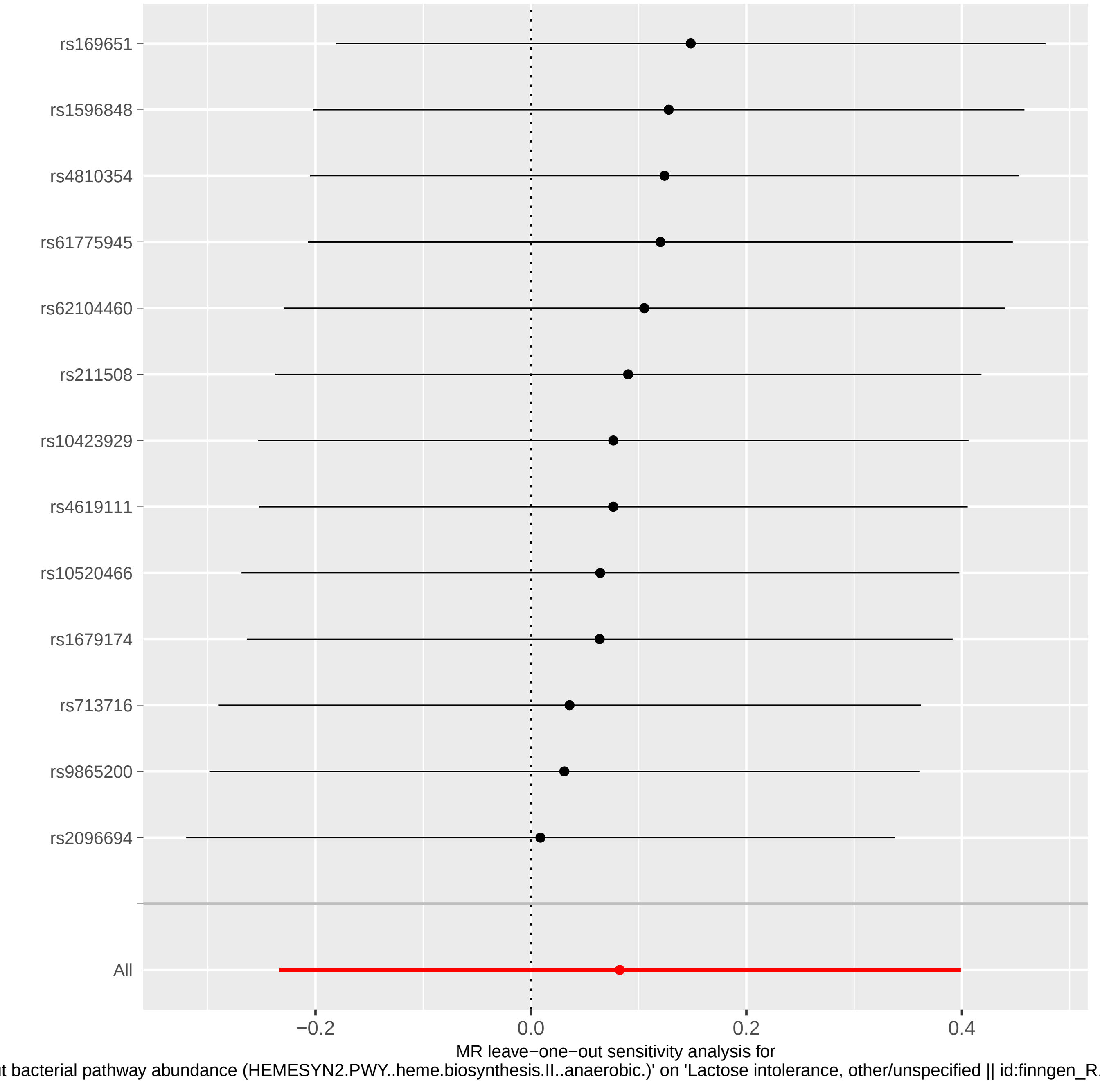

rs169651
rs1596848
rs4810354
rs61775945
rs62104460
rs211508
rs10423929
rs4619111
rs10520466
rs1679174
rs713716
rs9865200
rs2096694
All
−0.2
0.0
0.2
0.4
MR leave−one−out sensitivity analysis for
t bacterial pathway abundance (HEMESYN2.PWY..heme.biosynthesis.II..anaerobic.)' on 'Lactose intolerance, other/unspecified || id:finngen_R
